# Intraoperative Radiation Therapy: A Promising Treatment Modality in Head and Neck Cancer

**DOI:** 10.3389/fonc.2017.00148

**Published:** 2017-07-07

**Authors:** Lara Hilal, Karine A. Al Feghali, Paul Ramia, Ibrahim Abu Gheida, Jean-Pierre Obeid, Wassim Jalbout, Bassem Youssef, Fady Geara, Youssef H. Zeidan

**Affiliations:** ^1^Department of Radiation Oncology, American University of Beirut Medical Center, Beirut, Lebanon; ^2^Miller School of Medicine, University of Miami, Miami, FL, United States

**Keywords:** recurrent cancer, locally advanced, head and neck tumors, salivary gland tumors, intraoperative radiation therapy

## Abstract

Every year, almost 62,000 are diagnosed with a head and neck cancer (HNC) and 13,000 will succumb to their disease. In the primary setting, intraoperative radiation therapy (IORT) can be used as a boost in select patients in order to optimize local control. Addition of external beam radiation to limited volumes results in improved disease control over surgery and IORT alone. In the recurrent setting, IORT can improve outcomes from salvage surgery especially in patients previously treated with external beam radiation. The use of IORT remains limited to select institutions with various modalities being currently employed including orthovoltage, electrons, and high-dose rate brachytherapy. Practically, execution of IORT requires a coordinated effort and careful planning by a multidisciplinary team involving the head and neck surgeon, radiation oncologist, and physicist. The current review summarizes common uses, outcomes, toxicities, and technical aspects of IORT in HNC patients.

## Introduction

Head and neck cancers (HNCs) continue to take a high toll with an estimated incidence of around 62,000 new cases in the United States in 2016 (1). Radiation therapy (RT) is commonly used as adjuvant treatment or, as definitive modality when surgical resection is not possible. Delivering radiation at the time of resection of HNCs is particularly helpful in cases at high risk for recurrence, particularly where there is gross or microscopic residual disease or for recurrent disease (2). The safety and effectiveness of intraoperative radiation therapy (IORT) for HNCs have been established in studies from several institutions (3–5). Although IORT is mainly used in recurrent patients, few studies reported on its use in the primary setting. Two forms of IORT have been studied for HNCs: high-dose rate (HDR) brachytherapy (6) and external beam that includes electrons and orthovoltage photons IORT (3, 4). The purpose of this manuscript is to review experience over the past four decades with the use of IORT in patients with primary or recurrent cancer of head and neck.

## Role of IORT in Head and Neck Tumors

### Recurrent HNC

The treatment of recurrent HNC is challenging, especially in the setting of previous irradiation. As per the NCCN 2017 guidelines, surgery is the mainstay of treatment for resectable locoregional recurrences with or without the addition of postoperative reirradiation (7). However, the NCCN adds a word of caution that reirradiation should be limited to a highly selected group of patients (8). The main challenge in reirradiation is the dose limiting tolerance of surrounding normal tissue. Hence, many studies have reported on the use of IORT since it provides the advantage of decreasing the treatment volume to the site that is directly observed in the operating room (OR), in addition to the possibility of operative mobilization of organs at risk, and normal tissue shielding. Encouraging results have been observed with the use of IORT for recurrent head and neck tumors at the primary site, neck, or salivary glands (Table 1).

**Table 1:** Summary of retrospective studies on IORT use in recurrent head and neck cancer.

Reference	*N*^a^	Primary location (most common)	Median tumor size^e^	IORT location	IORT modality	Dose range	Median dose	Adjuvant therapy at rec.	Hx of RT	Duration to reirradiation	Median *F*/*U*	LC	Survival	Toxicity
Scala et al. (9)	*n* = 76 (87 sites)	Oral cavity (29%), SGT (18%), OP (16%)	Median field size: 5 = 6 cm	Neck (46%); face (13%)	HDR IORT	12–17.5 Gy^b^	12 Gy	24% EBRT (45 Gy) 41% (chemo)	EBRT: (59.8–63.9 Gy)	2 years	11 months	2 year IFLC: 62%	MS: 33 months (in field control) and 17 months (no control)	Flap revision: 4%, carotid hemorrhage: 1%, vagal neuropathy: 1%
Teckie et al. (10)	*n* = 57 (59 sites)	OP, hypopharynx, SGT	≤2 cm: 42% 2.1–4 cm: 32% >4 cm: 25%	Neck (71%); parotid (12%)	HDR IORT	12–20 Gy	15 Gy	21% EBRT: (50 Gy) 27% (chemo) 11% (both)	EBRT: median dose: 66 Gy	Median: 15 months	16 months	3-year IFPFS: 57%	3 year OS: 50% (in field control) vs. 32% (no control)	Fibrosis: 29%, trismus: 24%, cellulitis: 10%, CN injury: 26%, dysphagia: 39%, fistula: 15%
Zeidan et al. (11)	*n* = 46 (out of 96 total)	Parotid gland	≤2 cm: 47% 2.1–4 cm: 39% >4 cm: 14%	Parotid (100%)	IOERT (Mobetron)	15 Gy or 20 Gy	15–20 Gy	57% EBRT (45 Gy), 19% chemo	EBRT: median dose: 60 Gy	8.7 months	5.6 years	5 year RFS: 48.1% (recurrent)	3 year OS: 59%, 5 year OS: 48% (recurrent)^c^	Comp.: 27% vascular: 7%, trismus: 6%, ORN: 4%, fistulas: 4%, flap necrosis: 2%, wound dehiscence: 2%, neuropathy 1%
Zeidan et al. (12)	*n* = 198 (out of 231 total)	UAD tract	4.3 cm	Neck (100%)	IOERT	10–25 Gy	15–20 Gy	22% EBRT (45 Gy), 43% chemo	EBRT: dose not reported	NR	1.03 years	3 year RFS: 55%, 5 year RFS: 49% (for all)	3 year OS: 34%, 5 year OS: 26% (for all)	Vascular: 11.3%, fistula: 9.8%, wound dehiscence: 9.8%, neuropathy: 3%ORN: 4%
Perry et al. (6)	*n* = 34	Salivary gland (21%) and OP (21%)	≤2 cm: 53% 2.1–4 cm: 26% >4 cm: 21%	Salivary gland (21%) and OP (21%)	HDR-IORT	10–20 Gy	15 Gy	15% EBRT: (50 Gy) 21% chemo	EBRT: median dose: 63 Gy	median: 16 months	23 months	2 year LC = 56%	2 year OS = 55%, MS = 24 months	Fibrosis: 38, trismus: 23%, cellulitis: 14%, fistula or wound: 9%, ORN 3%, trigeminal neuralgia: 3%, 2nd tumor: 3%
Chen et al. (2)	*n* = 37 (out of 99)	SGT (100%)	≤2 cm: 28% 2.1–4 cm: 41% >4 cm: 30%	SGT (100%) parotid most common (34%)	IOERT	12–18 Gy	15 Gy	15% EBRT (54 Gy) 9% chemo	EBRT: median dose 60 Gy	3.1 years	3.7 years	5 year LC: 82% (with IORT) and 60% (without IORT)	3 year OS: 54%, 5 year OS: 34% (all). MS: 12 mo. (neck rec) vs. 20 months (primary site rec)	Superficial wound infection: 5%, trismus: 3%, Facial neuropathy: 3%
Chen et al. (4)	*n* = 137 d	OP, oral cavity, paranasal sinus, parotid.	≤2 cm: 45% 2.1–4 cm: 33% >4 cm: 22%	Local (64%); neck (28%); both (8%)	IOERT	NR	15 Gy	26% EBRT (54 Gy) 72% chemo	EBRT median dose: 64 Gy	13 months	18 months	3 year in field control: 62% 3 year LRC 51%	3 year OS: 36% (3-year OS: 44% primary rec compared with 19% neck rec)	Superficial wound infection: 3%, fistula: 1.5%, wound dehiscence: 0.7%, trismus: 0.7%, neuropathy: 0.7%
Pinheiro et al. (13)	*n* = 44 (34 SCC and 10 non-SCC^d^)	OP, oral cavity	NR	Skull base (56%) and neck (44%)	IOERT	12.5–22.5 Gy	NR	NR	NR	NR	6.3 years	2 year LF: 54% (SCC) and 48% (non-SCC)	2 year OS: 50% (non-SCC) and 32% (SCC)	Soft tissue: 11.3%, fistula: 6.8%, neuropathy: 11.3%, fatal hemorrhage: 2.2%, wound: 4.5%
Schleicher et al. (14)	*n* = 84 (113 sites)	Hypopharynx, larynx and OP	Median field size: 34 cm^2^	Jugular chain (80%)	IOERT	10–20 Gy	20 Gy	9.5%: chemo	EBRT: median dose: 56 Gy	median: 38.3 weeks	NR	LC: 24% R2, 41.7% R1, 50% R0	MS = 6.8 months	Wound healing: 9%, 4%, salivary fistula: 3.5%, necrosis: 2%
Nag et al. (15)	*n* = 38	Larynx and oral cavity	NR	Primary H&N site (29%), neck only (37%)	IOERT	15 Gy: close or microscopically + margins, 20 for gross	15 or 20 Gy	0% EBRT	EBRT: median dose 65.1 Gy	NR	30 months	2 year LC = 13%, 2 year LRC: 4%	2 year OS: 21%, 3 year OS: 8%	Comp.: 16%, orocutaneous fistula: 5%, fatal fistula, wound or tracheal dehiscence and carotid occlusion: 2.6% each
Martinez-Monge et al. (16)	*n* = 23 (31 total)	NR	NR	NR	IOERT	10–15 Gy	NR	NR	EBRT: median dose 50 Gy	NR	NR	2 year LRC: 26%: recurrent	2 year OS: 31% (recurrent)	Comp.: 10%
Ling et al. (17)	*n* = 25 (out of 30 total)	NR	NR	NR	IOERT	15 Gy	15 Gy	NR	NR	NR	30 months	3 year LRC: 60% (for all)	3 year OS: 70% (for all)	Comp.: 16%
Freeman et al. (18)	*n* = 52 (out of 75 total)	NR	>3 cm	Neck (100%)	IOERT	10–25 Gy	20 Gy	33% EBRT (Dose NR)	EBRT: dose NR	NR	2 years	2 year LC: 68%: all patients	2 year OS: 45% (all patients)	Comp.: 25% including carotid blowout, sepsis, ORN, PE, flap necrosis, MI and hypocalcemia
Toita et al. (19)	*n* = 17 (22 sites out of 24 total)	Oral cavity (46%)	NR	Neck (86%); primary (14%)	IOERT	10–30 Gy	20 Gy	67% EBRT (41.2 Gy)	EBRT: 26–70 Gy range	NR	19 months	2 year LC: 54% all^f^ GR: 0% MR: 55%, CM: 82%	2 year OS: 45%; 0% GR, 33% MR, 70% CM (all)	Comp: 22%, carotid blowout: 3 patients, osteoradionecrosis (all more than or = 20 Gy): 4 sites
Freeman et al. (3)	*n* = 64 (out of 104 total)	Mucosa of UAD tract (71%), SGT (23%)	NR	Neck (35%), skull base (19%), parotid (17%)	IOERT	15–20 Gy	20 Gy: neck, 15 Gy: skull base, oral cavity and SG	NR	NR	NR	2 years	2 year LC: 40% (all)	NR	ORN: 6%, fistulas: 6%, carotid blowout: 3%, MI and PE: 3%

*IORT, intraoperative radiation therapy; IOERT, intraoperative electron radiation therapy; HDR IORT, high-dose brachytherapy; F/U, follow-up; LC, local control; IFLC, in-field local control; IFPFS, in-field progression-free survival; RFS, recurrence-free survival; OS, overall survival; SGT, salivary gland tumor; OP, oropharynx; SCC, squamous cell carcinoma; Comp., complications; ORN, osteoradionecrosis; EBRT, external beam radiation therapy; RT, radiation therapy; LRC, locoregional control*.

*^a^N: patients with recurrent tumors who received IORT*.

*^b^12 Gy: negative margins, 15–17.5 Gy for microscopically positive margins*.

*^c^1% LR in the IORT field, 20% regional, 13% distant in all*.

*^d^It included primary and recurrent tumors*.

*^e^Median field size, when available, is given when median tumor size is not reported*.

*^f^LC: 78% primary and 43% for neck*.

#### Neck Recurrences

Intraoperative radiation therapy for neck recurrences is one of the most common IORT uses in head and neck tumors probably due to the difficulty of completely resecting recurrent tumors close to critical structures such as the carotid artery or due to fixation to deep tissues especially after fibrosis induced by previous irradiation. We previously reported one of the largest retrospective series on neck IORT (12); it included 231 patients with advanced cervical metastasis, 88% (198 patients) had recurrent tumors. All included patients had either microscopic or gross residual disease. Intraoperative electron radiation therapy (IOERT) with a median dose of 15–20 Gy was used. Postoperative EBRT was offered to 50 patients (21.6%). With a median follow-up of around a year, 5-year recurrence-free survival (RFS) and overall survival (OS) were 49 and 26%, respectively, for all included patients (12). Another study on neck IORT included 52 patients with recurrent tumors who received a median dose of 20 Gy of IOERT. With a median follow up of 2 years, 2-year local control (LC) and OS were 68 and 45%, respectively, for all 75 patients (18). Most of the other series on IORT for neck recurrences, listed in Table 1, included between 17 and 84 patients (10, 14, 15, 19). The majority used IOERT while two of the studies used high dose radiation brachytherapy known as HDR IORT. The median dose ranged from 12 to 20 Gy. Most used adjuvant EBRT in addition to IORT with a median dose range of 41–50 Gy. The 2-year in-field LC reached up to 62%. LC was higher in patients with gross total resection (GTR), where 2-year LC was 50–100% after GTR vs. 0–24% with gross residual disease. It was also higher for primary recurrences as compared to neck recurrences (LC: 100 vs. 75%, respectively). As for survival outcomes, in a study, by Teckie et al, on 57 patients who received 15 Gy of HDR IORT, 3-year OS reached 50% in patients who had in-field control vs. 32% in those not achieving in-field control (10). Survival was also superior for patients with clear margins; where 2-year OS reached 70% in patients with clear margins in a study by Toita et al. which included 17 patients with 22 treated sites (19). In summary, IORT, with a median dose of 15–20 Gy, results in very good LC in patients with neck recurrences and can improve survival outcomes, especially in those who attain negative surgical margins.

#### Primary Site Recurrences

Good outcomes have been also reported for recurrences at the primary site treated with IORT. Perry et al. presented a study on 34 patients that included salivary gland tumor (SGT) recurrences (21%) in addition to tumor recurrence at other head and neck the primary sites. Most of the patients had a history of irradiation with EBRT to a median dose of 63 Gy. Adjuvant EBRT at recurrence was given to 15% of the patients with a median dose of 50 Gy. With 10–20 Gy of HDR IORT, 2-year LC and 2-year OS were 56 and 55%, respectively (6). An earlier series by Nag et al. reported less favorable outcomes as compared to other listed studies. It included 38 patients, 29% of which were treated with IOERT for primary site recurrence. The dose was 15 Gy for close or microscopically positive margins and 20 Gy for gross disease. It is worth noting however that patients did not receive adjuvant EBRT in addition to IORT resulting in a 2-year LC and OS of 13 and 21%, respectively (15).

#### SGT Recurrences

Recurrent SGTs were addressed in two main studies, which included patients, treated with IOERT, with the parotid gland being the most common site. Patients in both studies had a history of irradiation (EBRT) with a median dose of 60 Gy. We reported on 46 patients with recurrent parotid tumors treated by 15–20 Gy IOERT in addition to EBRT in 54% (dose of 45 Gy) and chemotherapy in 19%. Favorable outcomes were observed with a relatively long follow-up of 5.6 years. For those with recurrent tumors, 5-year RFS and 5-year OS were both 48% (11). The second study included 37 patients with recurrent SGT who received a median dose of 15 Gy IOERT in addition to EBRT (54 Gy) in 15%. LC at 5 years was better with IORT compared to no IORT (82 vs. 60%) and 5-year OS was 34% for the entire sample (2).

#### Prognostic Factors

Most of the studies on IORT in the recurrent setting showed a significant correlation between in field control and margin status. Scala et al. reported that 1-year in-field control for patients with negative margins was 82% compared to 56% in those with a positive margin (9). At least five other studies also showed that positive margins (more so for gross residual than microscopic residual) at the time of IORT significantly predicted for in-field failure when compared to close or clear margins (2, 11, 13, 18, 19). In addition, doses of IORT of more than 15 Gy were shown to be associated with better LC (10, 12). Other prognostic factors for LC and RFS include pre-reirradiation recurrence-free interval of more than 12 months (10), use of adjuvant EBRT (9), absence of nodal extra-capsular extension (10), and tumor size (11). Furthermore, patients with neck metastasis who had no PNI, no LVSI, and no involvement of the carotid artery were reported to have better OS after IORT (12). Taken together, these results underscore the prognostic importance of surgical pathology details in addition to treatment dose in this patient cohort.

### Locally Advanced (LA) Primary HNC

One of the potential benefits of intraoperative RT in LA HNC is minimization of the time interval between surgery and RT as studies have shown that delayed radiotherapy compromises LC outcomes (20, 21). The importance of IORT in LA HNC is in boosting microscopic or gross residual disease in close proximity to or extending to critical structures, in a setting where negative surgical margins cannot be achieved without significant morbidity. A larger volume is usually irradiated postoperatively using external beam radiation therapy (EBRT). IORT has the advantage of reducing the volume of the radiation boost field, allowing dose escalation to target tissue with selective shielding of sensitive structures.

#### IORT As a Boost to Primary HNC

The Methodist Hospital of Indiana introduced IORT for HNC in 1982 to improve on LC rates and select patients’ survival outcomes (3, 22). Few studies have examined the role of IORT in the primary HN setting exclusively. Most studies included patients with recurrent HNC, and most included a heterogeneous patient population with a wide variety of HNC disease sites. One of the first published studies by Garrett et al. reported on 28 patients with LA or recurrent HNC treated with surgery followed by IORT with 1-year OS rates of 67%. Indications for IORT in this study were as follows: (1) gross residual disease, (2) microscopic residual disease, or (3) close margins. In this series, all patients with residual gross disease recurred locally, whereas LC rates were 87 and 75% for close surgical margins and microscopic residual disease, respectively. The 43% of the patients received EBRT, in addition to IORT, with a median dose of 50 Gy. Carotid blowout was found to be a major treatment complication (in two patients). Results were not stratified by primary versus recurrent treatment (22). The same group at the Methodist Hospital of Indiana also reported on their experience with IORT in 104 patients with LA and recurrent HNCs (40 patients previously untreated, and the rest with recurrent disease). Patients were treated with surgery followed by IORT at a dose of 15–20 Gy. Some of the indications for IORT use were close surgical margins, fixation to the carotid sheath, deep muscles of the tongue, pre-vertebral fascia, extension to skull base or dura, or preservation of critical structures function, such as facial nerve. The percentage of patients who also received EBRT in addition to IORT was not reported. Results were promising, with 2-year LC rates of 54%, with better LC rates for parotid cancers (*n* = 19, 2-year LC = 69%) and tongue cancers (*n* = 16, 2-year LC = 57%) (3). Another small study by Freeman et al. included 25 patients with primary (*n* = 11) or recurrent (*n* = 14) tumors close to the skull base, who were treated with IORT for close surgical margins, or residual gross or microscopic disease, with LC rates of 64% at 1 year. The 36% of those patients also received postoperative EBRT (23). A third paper by Freeman et al., mentioned in the above section on neck recurrences, that studied 75 patients with advanced cervical lymph node metastases with 2-year LC and OS of 68 and 45%, respectively, included 22 patients with primary advanced untreated disease. This study did not report on the percent of patients also receiving adjuvant EBRT (18). These three studies with promising results paved the way for more single-institution studies.

In a study by Pinheiro et al., 44 patients with recurrent (*n* = 31) and LA (*n* = 13) HNCs (56% with skull base cancers) were treated with IORT at doses between 12.5 and 22.5 Gy, with around 50% tumor control rates overall. All patients with primary LA tumors received adjuvant EBRT (dose not reported) after IORT (13). Similar control rates were also documented in another study including 25 patients with mainly primary (*n* = 17) LA HNC treated with surgery and 12 Gy of IORT, with 2-year locoregional recurrence-free survival and disease-free survival rates of 58.5 and 50.6%, respectively (21). Nag et al. also studied 53 patients with primary HNC (out of 65 included patients) who were treated with 7.5–20 Gy intraoperative HDR brachytherapy to sites inaccessible to intraoperative electron beam radiotherapy, with 5-year LC rates of 59% and 5-year OS rates of 42%, and no major intraoperative or postoperative complications (24). Although the abovementioned studies have limitations inherent to their retrospective design, they all report LC rates of 50–68% at 2–5 years. It is worth noting that the majority of those studies do not report on adjuvant EBRT use.

#### Primary SGTs

Locally advanced SGT may involve or be in close contact with vital nerves or blood vessels within the head and neck. Adequate surgical margins might be difficult to attain in such a context. IORT might therefore be a good option in patients with salivary gland cancers at high risk of recurrence. The largest report of IORT in the multimodal management of patients with parotid cancers is a single-practice experience, which included 96 patients with primary (50 patients) or recurrent (46 patients) parotid cancers treated between 1982 and 2007 (11). In this study, 5-year recurrence-free survival (RFS) rates were 77.8% and OS rates were 65.7% for patients treated in the primary setting with IORT boost dose of 15–20 Gy using 4–6 MeV electrons. Larger tumor size was predictive of recurrence after IORT, and patient age was predictive of survival on multivariate analysis. These results have to be put into perspective, as other RT modalities were tried in the management of LA parotid cancers with promising results. The use of fast neutron RT yielded 5-year locoregional control (LRC) rates of 92 and 63% in patients treated with RT alone (without surgery), and with postoperative RT for gross residual disease, respectively (25). Garden et al. also reported LRC of 85% with the use of postoperative EBRT in patients with malignant tumors of the parotid gland (26). Taken together, these studies demonstrate that IORT could be considered as an option in multimodal management of patients with primary HNC, to address gross or microscopic residual disease in a setting where complete resection would be too morbid due to proximity to a major vessel or critical nerve, or as a boost for traditionally radioresistant tumors, such as SGT.

## Toxicity of IORT

Intraoperative radiation therapy-related complications rate reported in the literature (Table 1) ranges between 22 and 52% in both the primary and recurrent setting (11–13, 18, 19, 27). Early studies by Toita et al. reported a significant increase in the rate of toxicities with doses exceeding 20 Gy (19), whereas other studies failed to show significant changes in toxicity rates with different doses (10, 12). Of the several reported complications, carotid artery rupture incidence ranged between 2 and 5% (3, 13, 18) and up to 10% in older series (19). Carotid blow out is a treatment complication associated with the highest mortality rates. Fistula/abscess rate ranged between 4 and 15% (3, 4, 10–12, 14, 18, 27). Wound related toxicity from cellulitis to flap necrosis ranged from 0 to 12% (2–4, 6, 10–12, 14, 27). Osteoradionecrosis rates are reported to range from 0 to 13% (3, 4, 6, 11, 12, 14, 19). Furthermore, some studies report on treatment related neuropathy ranging from 1 to 3% and mostly treated by symptomatic pain management (2, 4, 6, 11–13). Of note, higher neuropathy rates were noted in a study from MSKCC reporting the outcomes of 57 patients with recurrent tumors, where neuropathy rates reached 26%, trismus rate of 24%, and fibrosis rate of 29% (10). Similar rates of trismus and fibrosis, 28 and 23% respectively, were reported from MSKCC in another retrospective study of 34 patients with recurrent disease (6). It is worth mentioning, however, that the above studies used different toxicity scales and had variable median follow up. Taken together, IORT in experienced centers has reasonable toxicity profile, and does not increase perioperative mortality (2, 4, 5, 11, 12) or hospital length-of-stay (5).

## Radiobiology of IORT

Using IORT in different clinical settings, including HNCs, is well grounded in several well-known radio-biologic principles. IORT provides a significant dose–response relationship advantage with the high dose given in a single IORT fraction having 1.5–2.5 times the biological effectiveness of the same dose given at standard fractionation (28). While it is debatable to rely solely on the linear quadratic model at very high doses, there is little doubt that tumor cell survival is significantly reduced when using a higher dose per fraction as compared to conventional fractionation. Moreover, there remains a need to better evaluate the effects radio-sensitizing compounds may have on normal tissue tolerances as well as tumor cell survival in the IORT setting (29).

Despite the efforts made intraoperatively to decrease the treatment volume, provide adequate retraction, and adequate shielding, the high fraction dose that is used during IORT does not give ample time for normal tissue repair. This may contribute to vessel injury, neuropathies, fibrosis, and other late effects in surrounding healthy tissues (30, 31). However, the quick drop off in dose with IORT HDR may help prevent treating critical structures surrounding the post-op bed to high dose. Regardless of treatment modality (photons/electrons or brachytherapy), treating residual tumor cells with radiation during surgery as opposed to days or weeks postoperatively reduces the disadvantageous role of tumor cell re-population. Also, it is well established that ischemic tumor areas may be more resistant to radiation treatment due to the paucity of oxygen fixing DNA damage caused by free radicals, and thus, a single high IORT dose does not provide ample time for tumor re-oxygenation. However, it is quite likely that the effect of this unfavorable ischemic milieu is counterbalanced by the single high IORT dose, when compared to standard external beam fractionation (12, 31). Moreover, there is evidence to suggest that high dosage of radiation in a single fraction can eradicate cancer stem cells that would have been radioresistant at standard fractionation and has even been theorized to have implications in unleashing favorable immune responses such as the abscopal effect (32, 33). Therefore, IORT has a promising role from a radiobiology perspective, in improving LC after resection of primary or recurrent HNCs.

## Modalities for IORT

Intraoperative radiation therapy can be delivered using several techniques and modalities that optimize target dose while minimizing it to the surrounding tissues. HDR brachytherapy, and electron and photon IORT are methods for this localized delivery of dosage. While harboring several similarities, the physics and radiobiology involved generally display a broad variance among the modalities and allow for suitable selections tailored for different HNC patients. Employing these tools correctly plays a particularly critical role in HNCs where surgical management of certain territories may be constrained by essential tissues or adjacent vascular components.

High-dose rate IORT allows the administration of focused radiation in regions where an EBRT cone is not appropriate (34). Application consists of placing a high activity source in close physical proximity to the post-surgical tumor bed while retracting or shielding adjacent structures. Treatment times typically elapse 15–60 min, allowing for treatment during a surgical procedure in a shielded room (35). HDR IORT offers strict spatial restriction of the administered dose, owing to the sharp fall in the inverse square function at short distances from the HDR source, resulting in relatively very little dose delivered beyond the prescribed 100% isodose line (36).

Although HDR IORT offers several advantages in tumor bed management, electron (IOERT) or photon IORT on the other hand provide optimal flexibility for a wide range of treatment sites. These forms of IORT may be delivered using radiation at different energies (37). At the present time, external beam IORT is administered by a dedicated linear accelerator with parallel electron beams of 3–12 MeV kinetic energy or isotropic photon fields with energies between contact and superficial therapy of 50 kV X-rays (38). Such low photon energy beams (e.g., Intrabeam^®^ by Carl Zeiss AG, Germany) have demonstrated favorable outcomes in some sites but with many questions remaining unanswered (39, 40).

## Challenges and Future Directions in IORT for HNC

**Figure 1:**
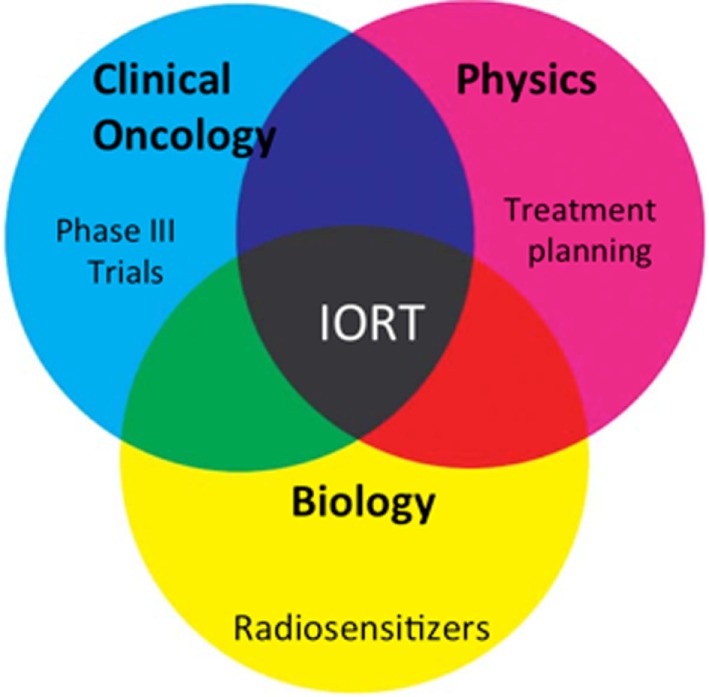
Summary of future directions. Phase III trials (clinical), treatment planning (physics), and radiosensitizers (biology) are at the forefront of current intraoperative radiation therapy research efforts.

Although IORT has emerged as a feasible modality in management of HNC, several challenges warrant further investigation. First, the efficacy of IORT needs to be further evaluated in randomized phase III trials. Due to paucity of radiotherapy centers with IORT equipment, this is best conducted *via* multicenter cooperative groups such as International Society of Intraoperative Radiation Therapy. Second, there needs to be professional guidelines describing IORT workflow and coordination between surgical and radiation specialties. Third, the therapeutic window for IORT in HNC patients needs to be further improved by using small molecule adjuncts that radiosensitize tumor cells and/or further protect normal tissues. Recent research efforts cast a promising future for HNC IORT (Figure 1) (28). For instance, the introduction of IOERT treatment planning system (41) affords accurate documentation of target and normal tissues dose distribution. This is anticipated to yield improved target coverage and better documentation of normal tissue doses.

Finally, assuring radiation safety when using IORT in the OR is a major concern. Typical stray doses from IORT at a 1 m distance from the patient are around 6 µSv per Gy of patient dose (42). Radiation safety regulations usually require shielding which reduces dose below the permitted constraints at any position around the OR under the assumption that the highest dose of stray radiation occurs in the direction of the beam during the complete workload. This need for structural shielding could be reduced by the possible use of mobile shielding walls (43). For example, in a study from Poland by Rabin et al., they calculated that it is sufficient to have mobile lead shield parameters of 1 cm × 140 cm × 150 cm between the accelerator in the OR and the control room to have the dose distribution in the patient plane meet radiation protection requirements (44). However, some authorities might object to mobile shielding due to the lack of control of correct placement by the personnel. A possible future solution might be the development of interlocked systems, which permit irradiation only if the crucial directions around the accelerator are adequately protected by correctly positioning the mobile shields (43).

## Conclusion

Intraoperative radiation therapy has emerged as an effective modality for HNC patients at high risk for local failure. To date, most of the scientific literature on head and neck IORT remains by and large limited to single institutional experiences. Recent technological advances and the advent of new IORT platforms predict an expansion of radiotherapy centers offering IORT. This is anticipated to accelerate opening of multi-institutional clinical trials in order to refine indications for IORT in HNC patients.

## Author Contributions

LH and YZ outlined the manuscript. LH added Table 1. LH, KF, IG, PR, J-PO, and YZ wrote the sections of the manuscript. FG, BY, and WJ reviewed and edited the manuscript.

## Conflict of Interest Statement

The authors declare that the research was conducted in the absence of any commercial or financial relationships that could be construed as a potential conflict of interest.
